# Yavar-70A, a novel water-in-oil adjuvant: A potency study in HPV-16E7d vaccine model

**DOI:** 10.22038/ijbms.2024.81654.17671

**Published:** 2025

**Authors:** Zeinab Mirzaei, Soyar Sari, Masoud Moghaddam Pour, Seyed Mehdi Hassanzadeh, Benjamin Damizadeh, Morteza Taghizadeh, Mehdi Mahdavi

**Affiliations:** 1 Department of Cellular and Molecular Biology, Faculty of Advanced Science and Technology, Tehran Medical Sciences, Islamic Azad University, Tehran, Iran; 2 Poultry Viral Vaccines Research and Production Department, Razi Vaccine and Serum Research Institute, Agricultural Research, Education and Extension Organization(AREEO), Karaj, Iran; 3 Department of BCG Vaccine Production, Production and Research Complex, Pasteur Institute of Iran, Karaj, Iran; 4 Department of Medical Vaccine, Razi Vaccine and Serum Research Institute, Agricultural Research, Education and Extension Organization (AREEO), Karaj, Iran; 5 Advanced Therapy Medicinal Product (ATMP) Department, Breast Cancer Research Center, Motamed Cancer Institute, Academic Center for Education, Culture, and Research (ACECR), Tehran, Iran; 6 Recombinant Vaccine Research Center, Tehran University of Medical Sciences, Tehran, Iran; # These authors contributed eqully to this work

**Keywords:** HPV-16-E7d, IFN-γ, Oil adjuvants, Vaccine formulation, Yavar-70A

## Abstract

**Objective(s):**

Adjuvants are some of the most important components used for vaccine formulation. In addition, the efficacy of vaccines is highly dependent on the nature of the adjuvants used. Therefore, new adjuvant formulations may help develop more potent vaccines. In the present study, the potency of an in-house and water-in-oil adjuvant (Yavar-70A) was compared with Montanide ISA 206 and Montanide ISA 266 in an HPV-16E7d vaccine model.

**Materials and Methods:**

Three HPV-16 E7d vaccines were formulated using three different adjuvants, Montanide ISA 206, Montanide ISA 266, and Yavar-70A, with standard protocols. Afterward, each formulation containing 10 μg of the E7d protein was administered thrice at two-week intervals to C57BL/6 mice. Serum levels of IFN-γ and IL-4 cytokines secreted from spleen cells, total IgG, and specific IgG1 and IgG2a isotypes were assessed using ELISA two weeks after the last immunization. Lymphocyte proliferative responses were also evaluated using the BrdU method.

**Results:**

The results indicated that the vaccine formulated using the Yavar-70A adjuvant showed the highest lymphocyte proliferation responses compared with other groups and higher IFN-γ cytokine release compared with that formulated using Montanide ISA 206. However, the vaccine formulated using Montanide ISA 206 induced the highest total IgG responses compared with other groups. Importantly, the vaccine formulated using Yavar-70A decreased IL-4 secretion compared with other vaccinated groups.

**Conclusion:**

The present study demonstrated that Yavar-70A induces cellular and humoral immunologic parameters against the HPV-16 E7d vaccine model comparable to commercialized oil-based adjuvants.

## Introduction

Human beings have always confronted various deadly diseases throughout humanity’s history, highlighting the need to find a solution to prevent such casualties. Vaccination is considered one of the most successful preventive methods to combat diseases (1). The history of vaccination began with investigations by Edward Jenner and continued with Pasteur’s research (2). Vaccination aims to induce protection against infections in which adjuvants have a critical role in vaccine formulations (3). Although vaccines have saved so many lives through decades and are a good field of research, some limitations, such as the lack of effectiveness or appropriate immune stimulation, hold back scientists from making more effective vaccines. Of great note, this setback was compensated by discovering adjuvants (4). The term “adjuvant” comes from the Latin word “adjuvare” which means help (5). Adjuvants were discovered in the 1920s by Ramon, who co-administrated some kinds of materials such as sugar and tapioca with tetanus and diphtheria toxoid vaccine and surprisingly observed that co-administration of such materials would be followed by augmentation of immune responses. Adjuvants can form a depot in the injection site, which helps vaccines be released gradually. Furthermore, some kinds of adjuvant can serve as a delivery vehicle that helps vaccine antigens reach immune-competent cells. Another characteristic that makes an adjuvant a useful partner of vaccines is their ability to induce T lymphocytes (6). Adjuvants can be used for different intentions, such as enhancing antigen immunogenicity, reducing antigen or immunization doses, and improving vaccine efficacy in newborns (7). Nowadays, it is accepted that adjuvants are a critical component in vaccine formulation (8, 9). Among adjuvants, oil adjuvants or adjuvant emulsions are often used in animals and humans. This class of adjuvants includes oil-in-water or water-in-oil emulsions such as the Montanide family and Freund incomplete adjuvant (FIA). The mechanism of this group is based on their ability to make depot and subsequently gradual release of antigens. These adjuvants, however, are not adequate for ordinary use due to their toxicity (10). Montanide, a famous family of this group, has been used in experimental vaccines such as human immunodeficiency virus (HIV) and malaria in different animals (11). Montanide has different types, including Montanide ISA 720, 51, 206, 266, etc. (12). Montanide 206 has been used in conjunction with the Borrelia anserina vaccine in laying chickens in 2013 (13) and during polyvalent snake antivenom production by *Waghmare et al.* in 2014 (14). ISA 266 is another type of Montanide used with the Gudair vaccine in order to develop protection against ovine Johne’s disease (OJD). Importantly, ISA 266 has been demonstrated to have the same efficacy as complete Freund’s adjuvant, presumably due to better antigen distribution during vaccination (15).

Here, we aimed to compare the activity of Yavar-70A, a water-in-oil-based adjuvant patented in Iran, with the same-structured Montanide ISA 206 and Montanide ISA 266 adjuvants in an HPV16E7d vaccine model for the first time.

## Materials and Methods

***Yavar-70A, Montanide ISA 206, and Montanide ISA 266 adjuvants***

The water-in-oil-based Yavar-70A adjuvant was developed by Zist Fanavar Pajoh Alborz Co. (Karaj, Iran) and patented in the Companies Registration Office and Industrial Property of Iran with patent No. 31952. All physical and chemical properties of Yavar-70A were characterized, and its safety pattern was determined in animal models (data not shown). In addition, Montanide ISA 206 and Montanide ISA 266 adjuvants were prepared by SEPPIC Company (France).

***Mice***

Six- to eight-week-old inbred female C57BL/6 mice were purchased from the Pasteur Institute of Iran (Karaj, Iran). The mice were housed for one week before the experiments in a standard animal house with a light/dark cycle (12 hr/12 hr) at 20-22 °C. All experiments on mice were carried out by an expert in accordance with the Animal Care and Use Protocol of the Islamic Azad University of Iran.

***Production and purification of recombinant HPV-16E7d***

 The recombinant HPV16E7d protein was expressed as a histidine-tagged protein in an *E. coli* BL21 (DE3) expression system and purified through Ni2+ affinity chromatography, as reported previously (16). After filtration, the sample was used for vaccine formulation and immunoassay analyses.

***Vaccine formulation***

In this case, HPV16E7d was prepared in Montanide ISA206, Montanide ISA 266, and Yavar-70A adjuvants. Briefly, the formulation was carried out at 30% to 70% of the antigen to the adjuvant with vigorous vortexes and subsequent homogenization. After vaccine preparation, 100 µl of the mixture containing 10 μg of the vaccine candidate was used for immunization.

***Experimental groups and immunization schedule***

The mice were divided into seven groups, each containing six mice (Table 1). Mice in groups 1 to 3 were subcutaneously immunized on day 0 with 100 µl of vaccine formulations containing 10 µg of HPV16E7d formulated using Montanide ISA266, ISA206, and Yavar-70A, respectively. In addition, the control groups were injected with Montanide ISA266, ISA206, Yavar-70A, and PBS, respectively, at the same time and route. All the experimental groups were boosted on days 14 and 28 with the same conditions.

***Lymphocyte proliferation assay***

Two weeks after the third immunization, the spleens from mice (n=6) in each group were removed under sterile conditions and suspended in sterile, cold PBS containing 2% FBS. Red blood cells (RBCs) were lysed with lysis buffer, and cell suspension was adjusted to 2×10^6^ cell/ml in RPMI-1640 (Gibco, Germany) supplemented with 10 % FBS, 4 mM L-glutamine, 1 mM sodium pyruvate, 100 µg/ml streptomycin and 100 IU/ml penicillin. Then, 100 µl of the cell suspension was dispensed into 96-well flat-bottom culture plates (Greiner, Germany) and stimulated with 10 µg /ml of the HPV16E7d. Phytohemagglutinin-A (Gibco, Germany), un-stimulated wells, and culture medium were used as positive, negative, and blank controls. All experiments were carried out in triplicate. After 72-hour cell culture, 20 µl of BrdU (Roche, Germany) was added to each well, and the plates were further incubated at 37 °C for 24 hr. After incubation, the plates were centrifuged at 300 g for 10 min, the supernatant was carefully aspirated, the plates were dried at 60 º C for 30 min, and 200 µl of Fixation/denaturation buffer was added to each well for 30 min. The plates were aspirated, and 1/100 of the anti-BrdU-HRP conjugate was added and incubated for 2 hr. Afterward, the plates were washed five times with PBS, and TMB (Tetramethyl benzidine) substrate was added to wells and incubated for 5 minutes in the dark at room temperature. The reaction was stopped by adding 2N H_2_SO_4_ (5% solution). The OD of each well was determined at 450 nm. The stimulation index (SI) was calculated using the following formula: SI = 450 OD of the stimulated wells /450 OD of the un-stimulated wells.

***ELISA of IFN-γ and IL-4 cytokines***

Two weeks after the second boosting, a total of 4×10^6^ spleen cells were placed on each well of the 24-well plates (Greiner, Germany) using complete RPMI-1640, stimulated *in vitro* with 10 μg/ml of the HPV16E7d and incubated at 3 °C in 5% CO_2_; un-stimulated samples were prepared in other wells. Three days post antigen recall, supernatants were removed, and the concentration of IFN-γ and IL-4 cytokines was estimated by quantitative mouse cytokine ELISA Kits (Mabtech, Sweden) according to the manufacturer’s instruction. The concentration of each sample (pg/ml) was calculated according to the standard curve.

***ELISA of specific total IgG antibodies and specific IgG1 and IgG2a isotypes***

Specific antibodies were determined by an optimized indirect ELISA. Briefly, 100 µl of HPV16E7d in PBS (10 µg/ml) was added into 96-well ELISA Maxisorp plates (Nunc, Naperville, IL) and incubated overnight at 37 °C. The wells were washed with PBS containing 0.05% Tween 20 (washing buffer) and blocked for 1 hour at 37 °C with 2% skimmed milk in PBS (blocking buffer). Plates were washed with washing buffer, and 100 µl of 1/100 to 1/12800 diluted sera was added to each well and incubated at 37 °C for 2 hr. The wells were washed five times with washing buffer and incubated for 2 hr with 100 µl of 1/10000 dilution of HRP-conjugated anti-mouse antibodies (Razi biotech, Iran). The wells were washed five times and incubated for 30 minutes with 100µl of TMB substrate in the dark. The reaction was stopped with 2N H_2_SO_4_, and color density was measured using an ELISA plate reader at OD 450 nm. In order to detect the specific IgG1 and IgG2a subclasses, 1/500 serum dilutions of mice were used, and detection was performed using goat anti-mouse IgG1 and IgG2a secondary antibodies (ISO-2 kit, Sigma, USA) according to the manufacturer’s instruction. 

***Statistical analysis***

All experiments were carried out in triplicate. All statistical analyses were carried out by Graph pad prism V 6.01 using the Mann-Whitney U test. The data are expressed as the means ± SD of each experiment. In all cases, *P*-values less than 0.05 were considered statistically significant.

**Table 1:** Experimental C57BL/6 mice groups and vaccine regimens for each group

Group No.	Vaccine regimen	Dose	No. of mice	Volume of injection	Route of injection
1	HPV16E7d-Montanide ISA-266	10 µg	6	100 µl	subcutaneous
2	HPV16E7d-Montanide ISA-206	10 µg	6	100 µl	subcutaneous
3	HPV16E7d-Yavar-70A	10 µg	6	100 µl	subcutaneous
4	Montanide ISA-266 control	-	6	100 µl	subcutaneous
5	Montanide ISA-206 control	-	6	100 µl	subcutaneous
6	Yavar-70A control	-	6	100 µl	subcutaneous
7	PBS control	-	6	100 µl	subcutaneous

**Figure 1:**
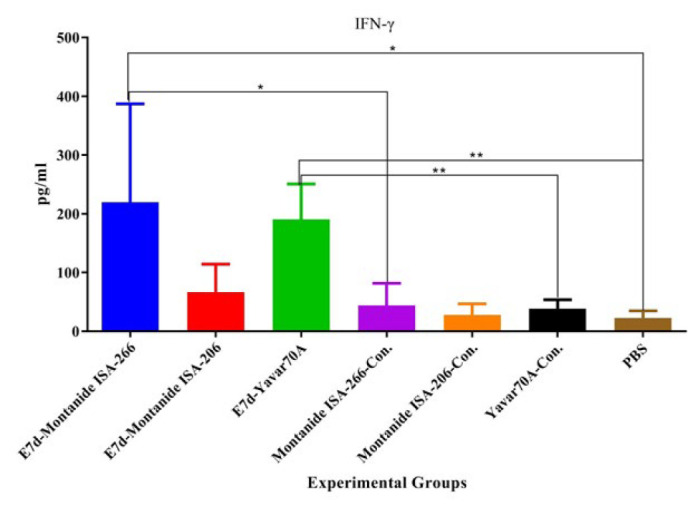
Results from lymphocyte proliferation in the experimental mice groups

Mice immunized with the recombinant HPV-16E7d protein formulated with the Yavar-70A adjuvant did not show a significant effect on lymphocyte proliferation as compared with those immunized with HPV-16E7d formulated with E7d-Montanide ISA-206 (P=0.0734) and E7d-Montanide ISA-266 (P=0.3176) groups.

**Figure 2:**
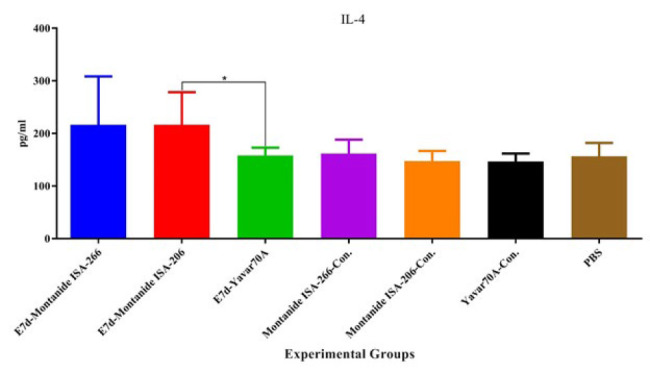
Results from the IFN-γ cytokine assay

Mice immunized with HPV-16 E7d formulated with Montanide ISA-266, Montanide ISA-206, and Yavar-70A increased IFN-γ cytokine response as compared with the corresponding adjuvant control groups (*P=0.0121, *P*=0.2571, and **P=0.0061, respectively). In addition, all the vaccinated groups increased IFN-γ release as compared with the PBS control group (E7d-Montanide ISA-266 and Yavar-70A-E7d versus PBS; **P=0.0061 and E7d-Montanide ISA-206 versus PBS; *P*=0.0667). Furthermore, mice immunized with the vaccine formulated with E7d-Yavar-70A significantly increased IFN-γ secretion compared to those immunized with the vaccine formulated with E7d-Montanide ISA-206 (**P=0.0029).

**Figure 3:**
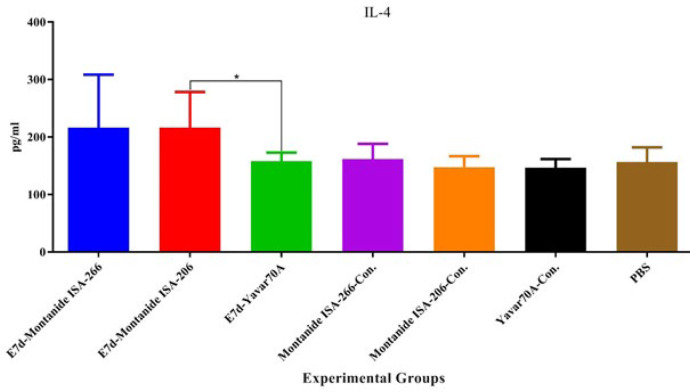
Results from IL-4 cytokine assessment

Mice immunized with the vaccine formulated with E7d-Yavar-70A significantly decreased IL-4 cytokine secretion compared to those immunized with the vaccine formulated with E7d-Montanide ISA-206 (*P=0.0350). In addition, mice immunized with the vaccine formulated with E7d-Yavar-70A did not show significant differences compared to those immunized with the vaccine formulated with E7d-Montanide ISA-266 (*P*=0.1748).

**Figure 4:**
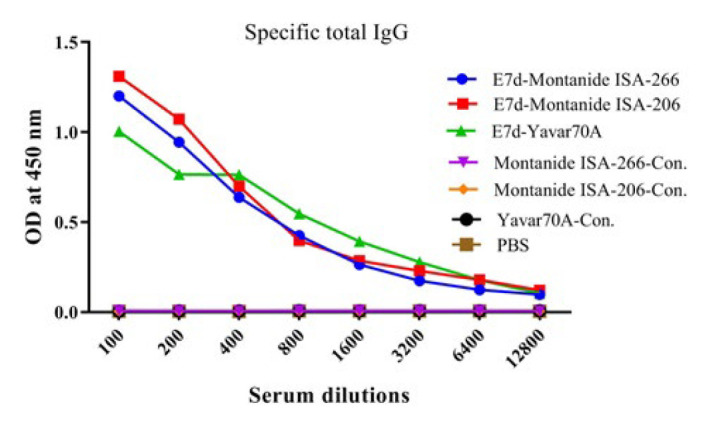
Results from the specific total IgG antibody in the experimental groups of mice

Mice immunized with the vaccine formulated with E7d-Montanide ISA-206 significantly increased the specific total IgG as compared with the vaccine formulated in E7d-Yavar-70A adjuvant at dilutions of 1/100 and 1/200 (**P=0.0034). In addition, mice immunized with E7d-Yavar-70A did not show significant differences compared with E7d-Montanide ISA-266 group in all serum dilutions (*P*>0.1839).

**Figure 5:**
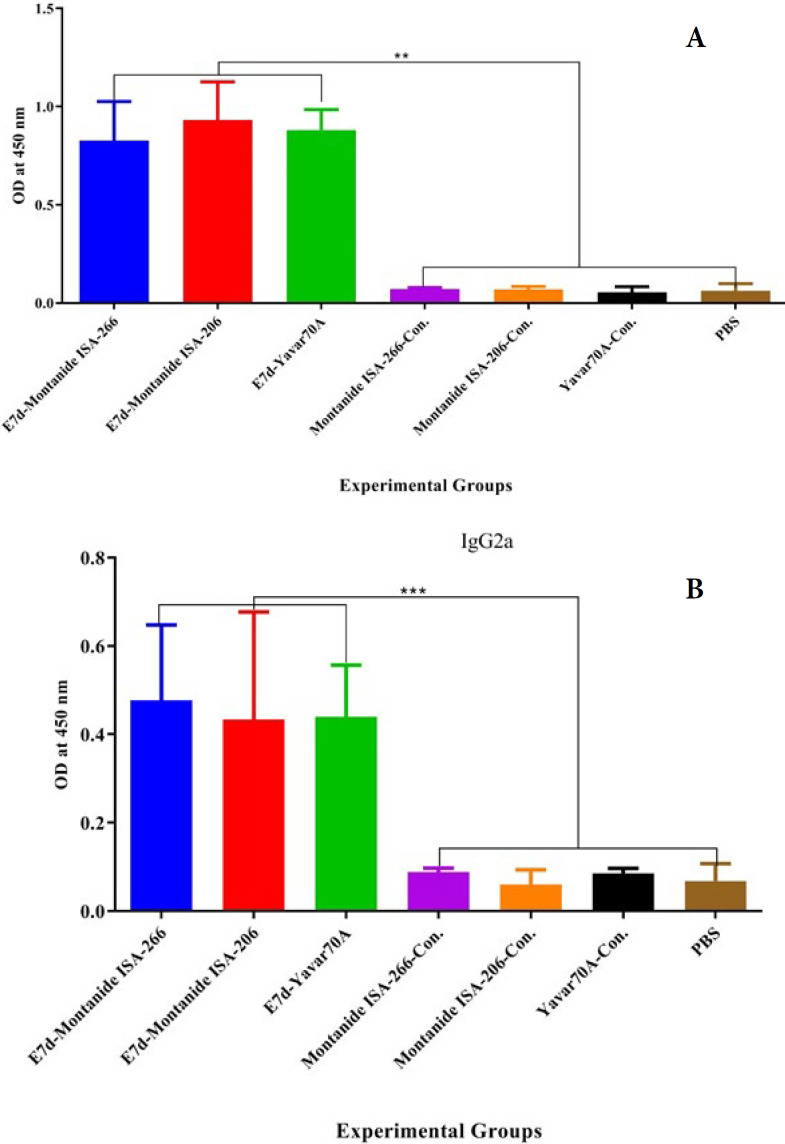
Assessment the specific IgG1 and IgG2a isotypes in the vaccinated mice

Results from the specific IgG1 (Figure 5A) antibody showed that mice immunized with E7d formulated using all three adjuvants (Montanide ISA 206, Montanide ISA 266, and Yavar-70A) significantly increased IgG1isotype as compared with the corresponding negative control groups (***P<0.0001). In addition, no significant differences were observed among the vaccinated groups (*P*>0.9445). Results from the specific IgG2a isotype (B) antibody showed that immunization with E7d formulated using Montanide ISA-206, Montanide ISA-266, and Yavar-70A adjuvants significantly increased the specific IgG2a isotype as compared with the corresponding negative control groups (**P<0.0055). Furthermore, no significant differences were observed among the three oil-based formulation vaccines (*P*>0.4701).

## Results

***Lymphocyte proliferation***

Results from lymphocyte proliferation according to the stimulation index showed that mice immunized with the E7d protein formulated using all three oil adjuvants significantly increased lymphocyte proliferation compared with all the control groups (*P*<0.0159). In addition, mice immunized with the E7d protein formulated using the Yavar-70A adjuvant did not show a significant effect on lymphocyte proliferation as compared with E7d-Montanide ISA-206 (*P*=0.0734) and E7d-Montanide ISA-266 (*P*=0.3176) groups (Figure 1).

***IFN-γ cytokine response***

 Results from the IFN-γ cytokine assay showed that mice immunized with E7d formulated using Montanide ISA266, Montanide ISA206, and Yavar-70A increased IFN-γ cytokine responses as compared with the corresponding adjuvant control groups (*P*=0.0121, *P*=0.2571, and *P*=0.0061, respectively). In addition, mice in the vaccinated groups increased the IFN-γ release as compared with the PBS control group (E7d-Montanide ISA-266 and Yavar-70A-E7d versus PBS; *P*=0.0061 and E7d-Montanide ISA-206 versus PBS; *P*=0.0667). Furthermore, mice immunized with E7d-Yavar-70A significantly increased the IFN-γ secretion compared to the E7d-Montanide ISA-206 group (*P*=0.0029). However, the E7d-Yavar-70A group showed no significant difference compared to the E7d-Montanide ISA-266 group (*P*=0.62) (Figure 2). 

***IL-4 cytokine assay***

 Results from the IL-4 cytokine assay showed that mice immunized with E7d-Yavar-70A significantly decreased IL-4 cytokine secretion compared with the E7d-Montanide ISA-206 group (*P*=0.0350). In addition, E7d-Yavar-70A did not show a significant difference compared with the E7d-Montanide ISA-266 group (*P*=0.1748) (Figure 3). 

***Specific total IgG antibodies***

Results from the E7d-specific total IgG antibody in the experimental groups showed that mice immunized with E7d-Montanide ISA-206 significantly increased the specific total IgG as compared with the E7d-Yavar-70A group at dilutions of 1/100 and 1/200 (*P*=0.0034). In addition, mice immunized with the E7d-Yavar-70A group did not show significant differences compared to E7d-Montanide ISA-266 in all serum dilutions (*P*>0.1839). Furthermore, mice immunized with the E7d-Montanide ISA-266 group did not show significant differences as compared with the E7d-Montanide ISA-206 group at all serum dilutions (*P*>0.6866) (Figure 4).

***IgG1 and IgG2a isotyping***

Results from the specific IgG1 (Figure 5A) antibody in the experimental groups showed that mice immunized with the E7d vaccine formulated using all three adjuvants (Montanide ISA 206, Montanide ISA 266, and Yavar-70A) significantly increased the IgG1 isotype as compared with the corresponding negative control groups (*P*<0.0001). In addition, no significant differences were found among E7d-Montanide ISA-206, E7d-Montanide ISA-266, and E7d-Yavar-70A vaccinated groups (*P*>0.9445). 

Results from the specific IgG2a isotype (Figure 5B) antibody showed that mice immunized with E7d formulated using Montanide ISA-206, Montanide ISA-266, and Yavar-70A adjuvants significantly increased the specific IgG2a isotype as compared with the corresponding negative control groups (*P*<0.0055). Furthermore, no significant differences were observed among the three oil-based formulation vaccines (*P*>0.4701).

## Discussion

Novel vaccines usually consist of purified antigens, considered safer than conventional non-purified antigens. Nevertheless, the main defect of such antigens is their weak immunogenicity. Therefore, adjuvants have become a good candidate for developing an effective vaccine (17). Oil adjuvants, especially the Montanide ISA family, are some of the most used categories of adjuvants formulated with different vaccines for animals and, more recently, humans (18). Montanide ISA-206 was used in the development of the polyvalent antiserum snake venom in a study carried out by *Waghmare et al.* (14). Montanide ISA-206 was also used in combination with CpG ODN in the FMDV A7 vaccine in a study performed by *Ren et al.*, demonstrating that the vaccine formulated with FMDV A7 antigen combined with Montanide ISA-206 and CpG ODN RW03, as a new CpG oligodeoxynucleotide, induced stronger immune responses and protective effects. They found that Montanide ISA-206 serves as a depot for the antigen, leading to the slow release of the antigen into the body, followed by a long-lasting immunity in animals (19). 

In this study, we developed an HPV-16 E7d vaccine formulated with an in-house and oil adjuvant Yavar-70A for the first time. We compared its function and activity with HPV-16 E7d vaccines formulated with Montanide ISA-206 and Montanide ISA-266 to induce cellular and humoral immunologic parameters in an animal model. Our findings indicated that Yavar-70A could stimulate lymphocyte proliferation as effectively as Montanide ISA-206 and Montanide ISA-266 adjuvants. This finding showed the potency of Yaver-70A in the induction of cellular immune responses at a comparable level with commercialized adjuvants Montanide ISA-206 and Montanide ISA-266. A study conducted on the Borrelia anserina inactivated vaccine formulated with Montanide ISA-206 in laying chickens showed that this adjuvant lacks the ability to induce cellular immune responses (13) appropriately. In this study, Yavar-70A showed a borderline superiority over Montanide ISA 206 but the same potency as Montanide ISA-266 in the induction of lymphocyte proliferation, an important sign of cellular immune response (20). Because cellular immunity is responsible for defending the body against intracellular pathogens such as viruses and some bacteria (21), this novel adjuvant can be a suitable choice against such pathogens due to stimulation of the cellular arm of the immune system and lymphocyte proliferation. Similar results were also observed in IFN-γ cytokine production in which mice immunized with the vaccine formulated using Yavar-70A significantly increased IFN-γ cytokine secretion as compared to those formulated with Montanide ISA-206; in contrast, comparable results were observed when comparing vaccines formulated with Yavar-70A and Montanide ISA-266. Interferon, a protein released when viruses invade a cell, can serve as an alarm, making the immune system produce proteins against pathogens (22). Interferon-gamma (IFN-γ) is known for its ability to regulate cellular immune system functions. IFN-γ can stimulate T CD8+ as well as Th1 lymphocytes that have the ability not only to help macrophages but can also play an important role in harnessing tumor growth and killing cells infected by bacteria, parasites, and viruses (23, 24). IFN-γ is the most important cytokine for switching Th1 and cellular immune response patterns, triggering T CD4+ and T CD8+ to invade viral and other intracellular pathogens (25). In the present study, mice immunized with the Yavar-70A-adjuvanted vaccine significantly increased IFN-γ secretion when compared with those immunized with the Montanide ISA-206-adjuvanted vaccine but showed the same responses as the Montanide ISA-266-adjuvanted vaccine; this means that a novel adjuvant, such as Montanide ISA-266, can be a more appropriate candidate for the induction of cellular immune responses compared to Montanide ISA-206. In the next step, we assessed the IL-4 cytokine as a Th2 index (26); our results showed the same Th-2 pattern response in mice immunized with vaccines formulated with Montanide ISA-206, Montanide ISA-266, and Yavar-70A. Th2 responses are followed by antibody production and humoral immune responses (27). The Th2 cytokine pattern and antibody responses are responsible for eliminating extracellular pathogens (28). Results from the specific total IgG showed that mice immunized with the Montanide ISA-206-adjuvanted vaccine increased total IgG at 1/100 and 1/200 serum dilutions compared to those immunized with the Yavar-70A-adjuvanted vaccine. On the other hand, no significant differences were observed between vaccines formulated with Montanide ISA-266 and Yavar-70A in the induction of specific total IgG. Various studies demonstrate the potency of the Montanide adjuvant family in the induction of humoral immune response (24, 29).

 Results from the specific IgG showed the superiority of Montanide ISA-206 in the induction of total IgG antibodies responses, while Montanide ISA-266 and Yavar-70A showed comparable responses in this parameter. Furthermore, results from IgG isotyping showed that all three adjuvants induced the same pattern of IgG1 and IgG2a isotypes without significant differences. Results from humoral immune responses confirmed the potency of Yavar-70A in the induction of humoral immunity, including both IgG1 and IgG2a isotypes; considering the specific function of each isotype, it could be concluded that a more bioactive humoral immune response occurred in all vaccinated groups. However, the present study did not assess the vaccine’s efficacy formulated using Yavar-70A versus the experimental tumor challenge. In addition, assessing more immunologic parameters is another limitation of this study that should be prospected in the future. 

## Conclusion

Considering all immunologic parameters, the results showed that Yavar-70A, as a novel candidate adjuvant, can induce the same immune responses as commercialized adjuvants Montanide ISA-206 and Montanide ISA-266, underscoring its potential for use in future vaccine formulations.

## Declaration

We have not used any AI tools or technologies to prepare this manuscript
